# Matrix materials as functional enablers in metabolite sensing: from mechanistic demands to translational potential

**DOI:** 10.1039/d6tb01338d

**Published:** 2026-10-30

**Authors:** Martin Varga, Guglielmo Risi, Saiyyna Stepanova, Mahsa Nasehi, Celal Bahtiyar, Emmanouil Rousakis, Lilian Witthauer

**Affiliations:** a Department of Diabetes, Endocrinology, Nutritional Medicine and Metabolism, Inselspital, Bern University Hospital, University of Bern Freiburgstrasse 3 3010 Bern Switzerland lilian.witthauer@unibe.ch; b Graduate School for Cellular and Biomedical Sciences, University of Bern Mittelstrasse 43 3012 Bern Switzerland; c Diabetes Center Berne Freiburgstrasse 3 3010 Bern Switzerland; d Department of Chemistry, Biochemistry and Pharmaceutical Sciences, University of Bern Freiestrasse 3 3012 Bern Switzerland; e Department of Digital Medicine, University of Bern 3010 Bern Switzerland

## Abstract

The performance of metabolite sensors is strongly shaped by the physicochemical properties of the matrix that governs analyte transport, interfacial stability, and signal coupling, yet the role of the matrix has largely been overlooked, often being viewed as a passive support rather than an active design parameter. This review analyses how matrix architecture controls sensing behaviour across polymeric, inorganic, carbon-based, and supramolecular material classes, examining mechanistic trade-offs between diffusion and binding, conductivity and biocompatibility, and structural stability and responsiveness in the context of electrochemical, optical, and electrical transduction modes. Particular emphasis is placed on operation in complex biological media, where protein adsorption, ionic fluctuations, mechanical mismatch, and dynamic metabolite flux impose constraints rarely captured in buffered laboratory systems. The comparative analysis reveals that no single matrix class universally optimizes sensitivity, selectivity, and stability; instead, sensor behaviour and performance-relevant characteristics emerge from alignment between the matrix architecture and the signal-generating mechanism. By framing matrix selection through these mechanistic principles, this review provides a foundation for rational matrix design toward continuous, multi-analyte, and clinically deployable metabolite sensors.

## Abstract 2

Matrix architecture governs analyte transport, interfacial stability and signal coupling. Tailoring the matrix to the transduction mechanism supports robust continuous sensing.

## Graphical abstract

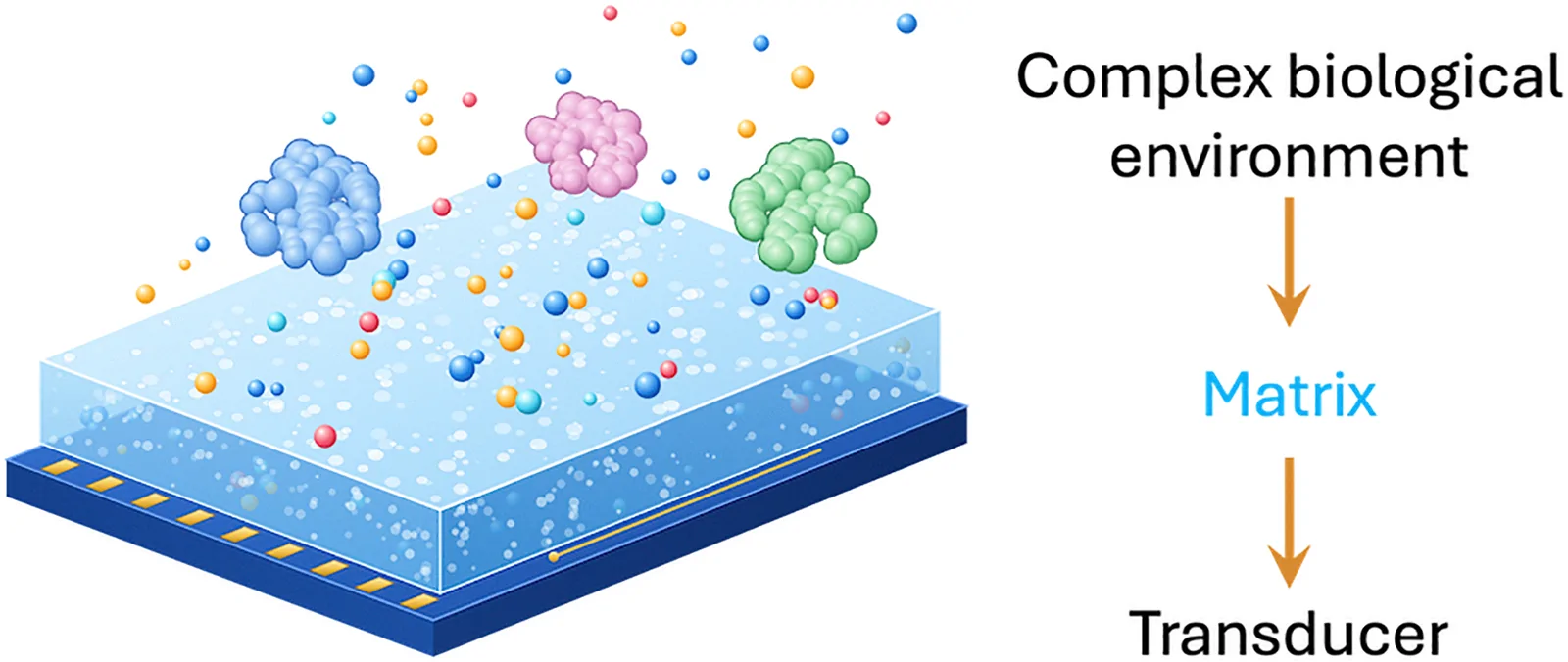

## 1 Introduction

The ability to monitor metabolites dynamically and continuously has become a central objective in precision medicine. In contrast to genomic or proteomic markers, metabolites provide an immediate functional readout of physiological state, reflecting enzymatic activity, metabolic flux, therapeutic response, and disease progression. Multi-analyte metabolic panels carry diagnostic and prognostic value across oncology, cardiology, endocrinology, and critical care,^1^ where continuous tracking of analytes such as glucose, lactate, ketone bodies, and pH informs sepsis detection, tumour metabolic reprogramming, diabetes management, cardiovascular risk stratification, and exercise physiology.^2–7^ Translating these insights into real-time clinical tools, however, requires sensing platforms capable of operating reliably in complex biological environments over extended periods.

Despite substantial progress in sensing technologies, most clinically deployed systems remain limited to single-analyte detection, with continuous glucose monitoring (CGM) representing the most prominent example. Multiplexed platforms capable of stable, long-term, *in vivo* metabolite tracking remain scarce. The challenge is not only one of sensitivity or selectivity at the transducer level, but of maintaining stable interfacial performance under continuous exposure to complex biological fluids.

Metabolite sensing platforms employ diverse transduction principles, including electrochemical, optical, field-effect transistor, calorimetric, and magnetoresistive mechanisms.^8–10^ These sensing technologies and their transduction mechanisms have been extensively reviewed in recent years, providing detailed insights into device architectures, signal generation strategies, and analytical performance. Each modality imposes distinct physicochemical requirements, such as optical transparency, electronic conductivity, dielectric stability, or electrochemical robustness. At the same time, the biological environment introduces chemical, mechanical, and biochemical constraints. Continuous exposure to biofluids leads to protein adsorption, ionic fluctuations, oxidative stress, and mechanical deformation, all of which progressively degrade interfacial integrity and signal fidelity. Without deliberate control of the material interface between the sensing element and its environment, long-term accuracy is difficult to sustain. This interface is defined by the matrix. Positioned between transducer and sample, the matrix governs analyte transport, interfacial reactions, microenvironmental conditions, and mechanical compatibility. It modulates diffusion, stabilizes recognition elements, suppresses nonspecific interactions, and determines the durability of the sensing architecture. A given material may simultaneously perform matrix functions and serve as transducer or recognition element; the present review focuses on its role as matrix, that is, on how it controls analyte transport, interfacial conditions, and signal coupling, regardless of what other roles it occupies in a particular architecture. Despite the central role of this material interface in shaping sensor behaviour, the matrix has often been treated as a secondary or supporting component in discussions of sensing technologies. Numerous biosensor reviews have been written that discuss, among other advancements, the exploitation of novel materials to induce stability, safety and biocompatibility. However, few focus on the matrix/biosensor interface, where the matrix is the sensing medium in which the core biosensing elements are embedded and with which they interact. This review defines this interaction and discusses the role of the matrix as a key design element that governs biosensor performance and, as such, should be taken into consideration as an optimization parameter during biosensor construction. In reality, the matrix sets the operating regime of the sensing architecture, determining which mechanisms limit response and which failure modes dominate under physiological conditions.

In this review, we examine sensing matrices as functional design elements in metabolite sensing. We analyze how matrix properties shape sensing behaviour across optical, electrochemical, and electrical sensing platforms, and compare the capabilities of polymeric, inorganic, carbon nanostructured matrices, and supramolecular material systems. These material platforms encompass hydrated polymer networks, rigid inorganic frameworks, electronically conductive carbon architectures, and affinity-driven supramolecular host–guest systems, each implementing matrix functions through distinct structural and physicochemical mechanisms. Particular emphasis is placed on how matrix architecture governs analyte transport, interfacial stability, and signal coupling in complex biological environments. By linking material design to sensing mechanisms and clinical requirements, this review aims to provide a framework for rational matrix engineering in next-generation metabolic sensor systems. Accordingly, the review does not aim to provide an exhaustive catalogue or ranking of metabolite sensors by analytical figures of merit. Quantitative performance metrics are included selectively where they clarify how matrix properties influence analyte transport, signal coupling, operational stability, or translational readiness.

## 2 Functional roles of matrices and signal coupling

From a systems perspective, the matrix mediates between the physicochemical requirements of the transduction mechanism and the chemical, mechanical, and biological constraints imposed by the sample environment.^8,9^ By regulating mass transport, interfacial interactions, and local environmental conditions, matrices decouple sensing chemistry from external perturbations including fouling, mechanical stress, ionic fluctuations, oxidative stress, and pH variation. This decoupling is particularly critical in continuous and *in vivo* metabolite sensing, where prolonged exposure to biofluids can rapidly degrade unprotected sensing interfaces.

### 2.1 Matrix functions

Matrices serve both passive and active roles in sensing architectures (Fig. 1). Passive functions include immobilization of sensing elements such as enzymes, antibodies, aptamers, dyes, and redox mediators;^11^ mechanical protection of fragile or chemically reactive interfaces under cyclic loading;^12^ and regulation of analyte transport through control of pore size, crosslink density, charge distribution, and chemical affinity.^13,14^ Effective immobilization preserves biological or chemical activity while maintaining analyte access; insufficient crosslinking risks leaching and signal drift, whereas excessive crosslinking reduces effective diffusion coefficients.^15^ The matrix further defines the local microenvironment for the sensing element by regulating hydration, ionic strength, and pH,^16^ which is essential for calibration stability in continuous platforms. In emerging sensing architectures, matrices increasingly assume active roles by incorporating redox mediators, conductive nanostructures, plasmonic elements, or dielectric components that directly participate in signal generation, amplification, or field modulation.^17^ Chemical stability under operational conditions is required across both roles: the matrix must resist hydrolysis, oxidation, redox-induced degradation, and structural rearrangement during continuous exposure to biofluids.^18,19^ Biofouling, defined as the nonspecific adsorption of proteins, lipids, and cells, further alters interfacial transport and can trigger inflammatory or foreign body responses that progressively isolate the sensing interface.^20^

**Fig. 1:**
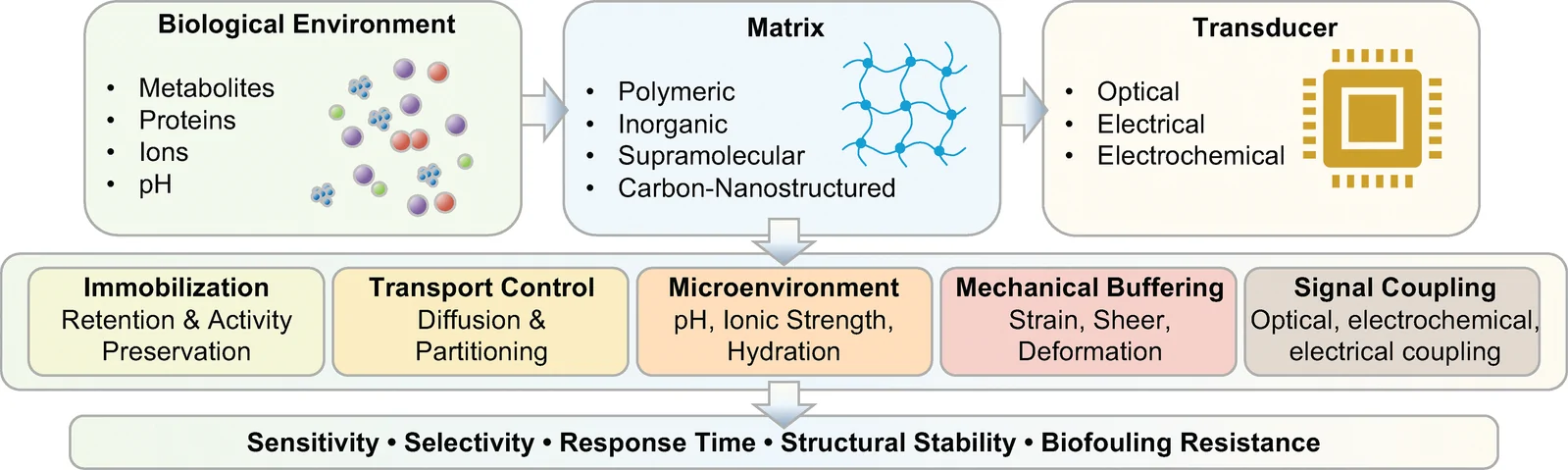
Functional roles of matrices at the interface between the biological environment and the transducer, and the sensor performance attributes they determine.

### 2.2 Transduction-mechanism dependence

Signal generation occurs at an interface whose properties are governed by the matrix, which therefore shapes how molecular-scale interactions are converted into measurable outputs. Matrix requirements are mechanism dependent. In optical platforms, the matrix becomes part of the electromagnetic pathway: signal may arise from fluorescence modulation,^21^ absorbance changes,^22^ colorimetry,^23^ surface-enhanced Raman scattering (SERS),^24^ interferometry,^22^ or refractive index shifts.^22^ Bulk optical transparency is necessary but not sufficient: the refractive index governs optical confinement, evanescent field penetration depth, and reflection losses, while nanostructured matrices modulate local electromagnetic enhancement in plasmonic systems. Autofluorescence and matrix Raman scattering set the noise floor, and photostability is critical under continuous illumination.^25^ In electrochemical platforms, the matrix forms part of the charge-transfer interface and is classifiable by measurement type as amperometric, potentiometric, conductometric, or impedimetric.^26^ Matrix thickness, porosity, and connectivity regulate analyte diffusion and therefore response time and signal magnitude; conductive matrix components reduce interfacial resistance and enhance sensitivity.^27^ Repeated potential cycling, local pH gradients, and ionic media exposure can induce structural degradation and baseline drift.^28^ In electrical and field-effect devices, including field-effect transistors (FETs),^29^ organic electrochemical transistors (OECTs),^8^ and chemiresistive sensors,^30^ the matrix defines the dielectric environment and screening behaviour. Ultrathin or highly hydrated coatings with controlled permittivity enhance field modulation; excessive thickness or ionic accumulation attenuates the electrical response.^31^ Across all three modalities, matrix properties directly influence signal amplitude, noise characteristics, drift, and temporal resolution, making matrix selection inseparable from transduction mechanism design.

### 2.3 Matrix-originated failure modes and design trade-offs

Operational failures in metabolite sensors can be traced to specific physicochemical mechanisms, including diffusion limitations,^13^ interfacial and structural instability,^18,19^ and perturbations of the local microenvironment.^16^ These mechanisms affect not only static performance metrics such as sensitivity and detection limit, but also dynamic performance, including response time, recovery time, hysteresis, reversibility, sensor lag, and drift under repeated concentration changes (Table 1). Modifications that alleviate one failure mechanism frequently introduce secondary trade-offs between transport efficiency, structural stability, and signal transduction.

**Table 1:** Failure mechanisms, manifestations, key material parameters, and mitigation strategies

Failure mechanism	Typical manifestation	Key material parameter	Mitigation strategies
Diffusion limitations	Increased response and recovery time; sensor lag; reduced sensitivity	Matrix thickness, pore size, porosity, crosslink density, swelling ratio	Reduce matrix thickness, increase porosity, introduce nanostructured pathways
Interfacial instability	Baseline drift, signal shift, signal loss, hysteresis	Swelling ratio, modulus mismatch, interfacial adhesion, immobilization chemistry	Crosslink stabilization, elastomeric composites, stronger interfacial adhesion, covalent immobilization; stronger encapsulation
Microenvironmental perturbation	Sensitivity decay, altered selectivity, nonlinear response	Water content, buffering capacity, charge density, ion permeability	Buffered matrices, ion-regulating layers, controlled oxygen transport

## 3 Matrix material platforms

The functional roles of matrices outlined above are determined by the physicochemical properties of the material constituting the sensing interface. Parameters such as polymer chain architecture, pore size distribution, surface chemistry, charge density, and electronic structure directly influence analyte transport, recognition stability, interfacial reactions, and signal coupling.^8,32,33^ While matrix functions can be described in terms of transport regulation, microenvironment control, and transduction efficiency, their implementation depends on the intrinsic characteristics of specific material classes. In this section, major material platforms are discussed with respect to how their structural and chemical properties enable the performance-defining roles described previously. Rather than presenting materials descriptively, the focus is placed on the relationship between material properties and sensing behaviour, including diffusion kinetics, interfacial stability, resistance to fouling, and compatibility with electrochemical, optical, or electronic transduction mechanisms. Representative matrix platforms are summarized in Fig. 2.

**Fig. 2:**
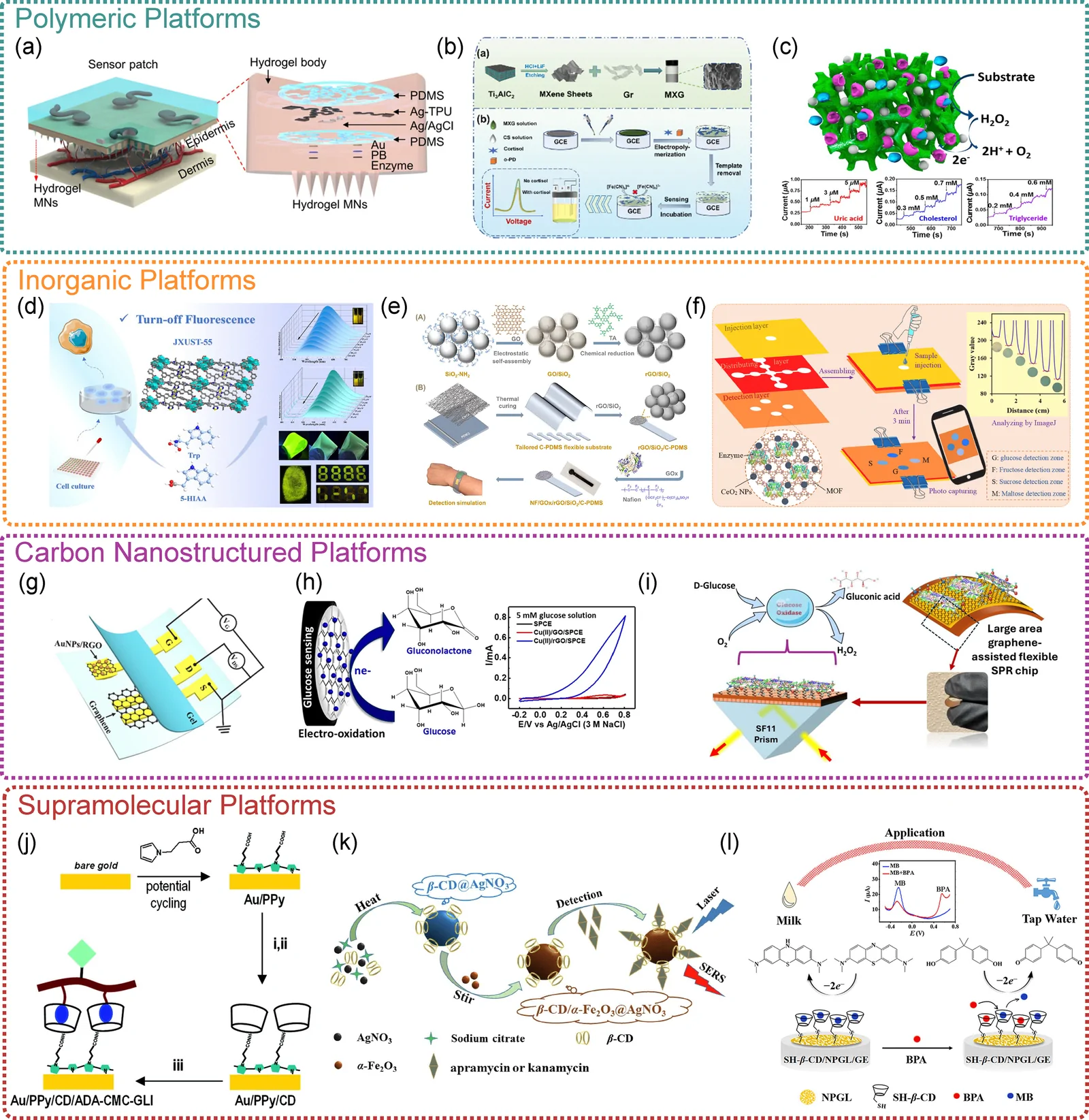
Representative matrix platforms across polymeric, inorganic, carbon nanostructured, and supramolecular systems. (a) Methacrylated hyaluronic acid hydrogel microneedles for ISF extraction and transport control.^34^ (b) MXene/graphene-based molecularly imprinted polymer with selective binding sites.^35^ (c) Conductive polyaniline hydrogel for multiplex electrochemical sensing.^27^ (d) Eu(iii)-MOF (JXUST-55) enabling analyte-induced luminescence quenching.^36^ (e) rGO/SiO_2_ nanohybrids in deformable glucose-sensing platforms.^37^ (f) CeO_2_ @Fe-MOF paper-based cascade biosensor for colorimetric saccharide detection.^38^ (g) Graphene-based electrolyte-gated transistor sensor with AuNPs/rGO gate.^39^ (h) Cu(ii)/rGO nanocomplex-modified electrode for enhanced electrochemical sensing.^40^ (i) Graphene-enhanced flexible SPR sensor.^41^ (j) Cyclodextrin-based multilayer assemblies for structural organization.^42^ (k) *β*-CD/*α*-Fe_2_*O*_3_ @AgNP SERS matrix for analyte capture and signal enhancement.^43^ (l) Cyclodextrins immobilized on gold electrodes for host-guest sensing.^44^ Adapted with permission from ref. 34 (a), ref. 35 (b), and ref. 27 (c). Copyright 2023, 2023, and 2015 American Chemical Society, respectively. Adapted with permission from ref. 36 (d) and ref. 37 (e). Copyright 2026 and 2021 American Chemical Society, respectively. Adapted with permission from ref. 41 (i). Copyright 2025 American Chemical Society. Adapted from ref. 38 (f), ref. 39 (g), ref. 43 (k), and ref. 44 (l) with permission from Elsevier. Adapted from ref. 40 (h) under Creative Commons CC BY 4.0. Adapted from ref. 42 (j) under Creative Commons CC BY 3.0.

### 3.1 Polymeric matrices

Polymeric matrices represent a widely applied material class in metabolite sensing. Their network architecture, defined by chain composition, crosslink density, and functional side groups, enables controlled adjustment of diffusional transport, hydration state, and interfacial chemistry.^32,33^ Mechanical compliance can additionally be tuned to reduce stress at the tissue interface. How these parameters shape sensing performance is transduction-dependent. In enzymatic electrochemical glucose sensors, matrix composition regulates substrate diffusion to glucose oxidase (GOx), mediator mobility, and long-term enzyme stabilization, thereby defining response time, linearity, and operational stability.^27,45,46^ This is directly reflected in commercial CGM systems: in oxygen-dependent GOx, crosslinked polyurethane (PU)- or poly(ethylene glycol) (PEG)-based enzyme matrices are combined with diffusion-limiting membranes to balance glucose and oxygen transport,^47,48^ whereas mediator-based sensors employ redox-polymer matrices that electrically wire the enzyme to the electrode.^49,50^ In optical oxygen sensors, sensitivity and response kinetics depend on oxygen permeability and diffusivity within the host polymer in addition to luminophore photophysics.^22,51^

Across sensing modalities, polymeric matrix design involves inherent trade-offs. Increasing crosslink density enhances structural stability but reduces effective diffusion coefficients. High loading of functional additives can improve signal amplitude yet introduce transport limitations or mechanical fragility. In implantable and minimally invasive formats, additional constraints arise from biofouling, dehydration, and mechanical mismatch at the tissue interface. Consequently, polymer selection is not a secondary material choice but a central design parameter that defines sensing kinetics, stability, and long-term functionality.

#### Hydrogels

Hydrogels are crosslinked polymer networks whose equilibrium swelling, governed by the balance between osmotic and elastic forces (Flory-Rehner theory), defines mesh size, polymer volume fraction, and effective analyte diffusivity.^32,33^ Common systems include PEG diacrylate (PEGDA), selected for low protein adsorption and adjustable crosslink density;^52^ polyacrylamide (PAAm), providing mechanical robustness and high hydration; and poly(*N*-isopropylacrylamide) (PNIPAAm), which introduces temperature-dependent phase transitions near physiological conditions.^53–56^ Across these systems, analyte transport depends on the relation between hydrodynamic solute size and mesh size: increasing crosslink density reduces mesh size and effective diffusion coefficients, improving dimensional stability but increasing response time.^52^ PEGDA and PAAm networks are frequently processed into microneedle geometries, where crosslink density must simultaneously ensure insertion strength and predictable diffusion for interstitial fluid (ISF) sampling.^34,57–59^ Beyond serving as permeable hosts for enzymes, fluorophores, or synthetic receptors, hydrogels can function as active transduction elements in which changes in volume, refractive index, or mechanical modulus are converted into optical or electrical signals.^33^ Stimuli-responsive variants couple analyte-responsive functional groups to network phase behaviour, amplifying molecular recognition into macroscopic signals but introducing hysteresis and delayed response kinetics from bulk network rearrangement.^33,60^ Integration of conductive polymers or ionic pathways can decouple mechanical compliance from electrical transduction,^45,46^ yet hydrogel matrices remain inherently susceptible to swelling-induced drift, structural fatigue, and biofouling under continuous *in vivo* operation.^61^

#### Thin-film and interfacial polymer matrices

Unlike bulk hydrogels, where mesh size and swelling thermodynamics dominate transport, thin polymer films operate in a confinement regime where analyte sorption and diffusion across a defined thickness determine response characteristics. Polymers such as poly(methyl methacrylate) (PMMA), poly(ethyl methacrylate) (PEMA), poly(vinyl alcohol) (PVA), and polystyrene (PS) are commonly employed because their refractive index, polarity, and free volume can be tuned through composition and processing.^62–64^ In optical platforms, thin coatings modulate the evanescent field or interferometric cavity through changes in refractive index, dielectric constant, or film thickness upon analyte sorption.^65^ This is exemplified in phosphorescence-based oxygen sensors, where Stern–Volmer constants, humidity sensitivity, and photostability vary with polymer chemistry: acrylate matrices such as poly(propyl methacrylate) (PPMA) and PEMA differ in permeability and baseline stability from sol–gel systems, reflecting variations in free volume, hydrophobicity, and oxygen diffusivity.^66,67^ Diffusion through both the sensing layer and protective overlayers can dominate response time, underscoring that interfacial architecture rather than indicator chemistry governs dynamic behaviour. Material polarity determines application scope. Hydrophilic PVA exhibits swelling-mediated refractive index modulation suited to vapor- and breath-phase sensing, where analyte-induced plasticization alters cavity length or effective optical path,^68^ while hydrophobic PMMA and PEMA provide controlled volatile organic compound sorption and reduced water interference. In surface plasmon resonance (SPR) configurations, thin polymer layers extend the sensing volume beyond the evanescent decay length, increasing analyte loading while maintaining optical confinement.^53^ PEG-based antifouling overlayers suppress non-specific protein adsorption^69^ but excessive thickness attenuates field-based transduction through dielectric screening. Across these systems, film thickness, permeability, and optical properties function as co-dependent rather than independently adjustable design variables.^66^

#### Molecularly imprinted polymers (MIPs)

In MIPs, selective binding sites are encoded into a crosslinked polymer network by polymerizing around a template molecule; its removal generates cavities complementary in size, geometry, and functional group orientation to the target analyte.^70–72^ This integrates molecular recognition directly into the matrix, where imprinted cavities act as selective partitioning domains that enrich the target while suppressing structurally related interferents,^73,74^ enhancing sensitivity particularly in electrochemical and impedance-based configurations. However, increased crosslink density improves cavity fidelity but reduces effective diffusivity, tightly coupling selectivity to response time. This trend is generally observed across polymers and hydrogels. In a study of poly(2-hydroxyethyl methacrylate) (PHEMA) networks a systematic increase of crosslinker concentration from 1 to 10 wt% shows that the diffusion coefficient decreases by approximately six-fold, while the equilibrium swelling ratio changes only from 1.7 to 1.5.^75^ Another study employing dextran methacrylate (Dex-MA) hydrogels reports an approximately doubled glucose sensor response time from 29.1 ± 1.5 to 55.3 ± 3.2 s upon increase of crosslink density, consistent with reduced glucose diffusivity through the more densely crosslinked network.^76^ MIP performance is transduction-dependent. In electrochemical systems, electropolymerized films on electrode surfaces modulate charge transfer resistance or redox current upon analyte binding,^74,77,78^ where film thickness, porosity, and template removal efficiency govern interfacial impedance, and excessive thickness can attenuate signal despite high selectivity.^79^ In optical platforms, analyte uptake alters refractive index, fluorescence, or Raman scattering. In the heat-transfer method, glucose binding to a MIP layer alters the interfacial thermal resistance and shifts the measured temperature at constant heating power.^80^ MIPs are frequently combined with conductive nanomaterials, plasmonic particles, or redox mediators to overcome the insulating polymer backbone.^35,81^ In such hybrids, it is often the nanostructured composite rather than the imprinting process that governs sensitivity, a distinction relevant when evaluating ultrasensitivity claims for low-abundance metabolites. The contribution of each component can be isolated by testing the MIP without the nanomaterial and the nanomaterial-loaded non-imprinted polymer (NIP) without imprinting. A clear example is the gold nanoparticle (AuNP)–MIP hybrid glucose sensor by Sehit *et al.* The MIP without AuNPs showed no detectable glucose response over 1.25 nM–2.56 µM, whereas the AuNP–MIP responded logarithmically over 1.25–320 nM, with 1.25 nM as the lowest concentration detected, also in 100-fold diluted human serum. Comparative rebinding experiments at 10–40 nM glucose showed signal suppression at the AuNP–MIP but negligible signals at the AuNP–NIP, confirming that glucose recognition arises from the imprinted cavities.^82^ Despite their chemical robustness relative to biological receptors,^83^ MIP matrices face translational constraints: binding site heterogeneity, incomplete template removal, and non-specific adsorption in complex biofluids reduce effective selectivity.^73,84^ Imprinting small, highly polar metabolites such as glucose or lactate remains challenging due to weak pre-polymerization interactions and competitive hydrogen bonding with water. Surface and oriented imprinting strategies address accessibility and binding homogeneity,^85^ but long-term performance data under physiological conditions remain limited.

#### Conductive polymers

Conductive polymers introduce mixed ionic-electronic transport into polymer matrices, enabling direct coupling between biochemical reactions and electrical transduction. Unlike insulating matrices, conjugated polymers such as poly (3,4-ethylenedioxythiophene) (PEDOT), polyaniline (PANI), and polypyrrole (PPy) participate actively in signal formation through redox-mediated doping and dedoping.^46,86^ Ion penetration into the hydrated polymer bulk modulates charge carrier density along the conjugated backbone, allowing the matrix to function as a volumetric transducer.^87^ This is exploited in OECTs, where the conductive polymer channel serves as both recognition interface and amplification element.^88^ In enzyme-functionalised PEDOT-based OECTs, metabolite-driven redox reactions alter the channel doping state, producing conductance changes that scale with analyte concentration,^89^ and n-type accumulation-mode OECTs have achieved mediator-free lactate detection through direct electron transfer between lactate oxidase (LOx) and the polymer channel.^90^ Material-specific doping chemistry further shapes sensing behaviour. PANI exhibits proton-coupled conductivity intrinsically sensitive to local pH fluctuations, exploitable in enzyme-based sensing where redox turnover generates protons.^86,87^ PPy, while readily electropolymerized and biocompatible, is prone to brittleness and overoxidation during repeated cycling, limiting long-term stability. PEDOT and PEDOT:poly(styrene sulfonate) provide low interfacial impedance in aqueous media, improving electronic coupling to ionic biofluids, though swelling and delamination constrain long-term operation.^46^ Conductive polymer hydrogels extend these properties into three-dimensional matrices: hierarchically nanostructured networks have enabled detection of uric acid and cholesterol by combining enhanced analyte diffusion with continuous electronic pathways and low interfacial resistance.^27,86^ Signal transduction in these systems relies on reversible redox doping and volumetric ion exchange, making them inherently susceptible to overoxidation, structural rearrangement, and swelling-driven conductivity drift during continuous operation.^87,90^ Long-term reliability therefore requires coordinated control of redox stability, ionic permeability, and mechanical robustness.

### 3.2 Inorganic matrices

Inorganic sensing matrices derive their function from structurally defined frameworks whose pore geometry, surface crystallography, electronic band structure, and defect chemistry are established during synthesis. These materials remain substantially more stable than polymeric or biological counterparts,^91,92^ though hydrolytic degradation in certain metal–organic frameworks (MOFs) imposes material-specific constraints.^93^ Unlike polymeric matrices, where chain mobility and hydration-dependent swelling continuously modulate transport,^94^ inorganic frameworks engage analytes through directionally constrained interactions: chemisorptive bonds at metallic surfaces, coordinative binding at Lewis acidic sites, and size- and charge-selective molecular sieving.^91,92,95^ This geometry-governed stability comes at a cost: the orders-of-magnitude modulus mismatch between inorganic phases (GPa) and biological tissue (kPa–MPa) impedes conformal mechanical coupling, promoting delamination, micromotion artifacts, and signal drift.^96^ Moreover, no single inorganic material simultaneously provides the conductivity, biocompatibility, selectivity, and antifouling resistance required in complex biofluids; performance therefore emerges from composite architectures where each component addresses a specific limitation.^47,97^ The four classes discussed here, MOFs,^92,98^ silica-based networks,^91^ metallic matrices,^95^ and metal oxide systems,^47^ illustrate how distinct combinations of porosity, electronic structure, and surface chemistry yield different sensing modalities. Across all four, a common trend can be observed: while the surface reactivity advantageously enables sensitive analyte recognition, it simultaneously renders the interface vulnerable to competitive adsorption, fouling, and degradation in protein-rich media. Related architectures, including MOF-derived porous carbons and molecularly imprinted silica, are omitted here as they are discussed in the context of carbonaceous and polymeric matrices, respectively.

#### Metal–organic frameworks

Although sometimes classified as organic–inorganic hybrids, MOFs are included here. MOFs are highly porous crystalline materials constructed from inorganic metal nodes and organic linkers assembled through reticular synthesis.^99,100^ Surface areas exceeding 7800 m^2^ g^−1^ and tunable pore dimensions from sub-nanometer to mesoporous regimes enable precise control over analyte transport and molecular confinement.^101–103^ Framework topology, linker functionality, and coordination chemistry collectively determine pore aperture, internal surface chemistry, and charge distribution, thereby regulating host–guest partitioning, diffusion kinetics, and sensing element loading density. Functionalisation of linkers with amino or nitro groups, whether through direct incorporation or post-synthetic modification, modulates binding affinity and selectivity,^104^ while accessible Lewis acidic metal sites provide coordination-based recognition of small metabolites and ions. As many MOFs exhibit limited intrinsic electronic conductivity,^105,106^ electrochemical configurations require incorporated redox mediators or conductive hybrid components. Examples include UiO-66-NH_2_ with electroactive dye tags for signal amplification,^107^ boronic acid-functionalised MIL-100(Cr)-B enhancing enzyme immobilization *via* hierarchical porosity,^108^ and Ag nanoparticles within Ni-MOF matrices providing conductive pathways for electron transfer.^109^ Across these configurations, the MOF serves not as the electrode but as a high-surface-area concentrator that co-localizes analyte, mediator, and enzyme within diffusion distances shorter than those achievable by surface immobilization alone. MOFs also support optical sensing through framework-integrated luminescence originating from π-conjugated linkers, lanthanide f–f transitions, or emissive guest species.^110,111^ Host–guest interactions alter excited-state dynamics through photoinduced electron transfer, ligand-to-metal or metal-to-ligand charge transfer, and Förster or Dexter energy transfer, with analyte coordination to open metal sites or functionalised linkers perturbing local electronic environments to produce fluorescence quenching or enhancement. Eu^3+^-doped MOF platforms have demonstrated detection of 5-hydroxyindoleacetic acid (5-HIAA) and related metabolites through analyte-induced fluorescence quenching in biological fluids.^36,112^ Ratiometric strategies incorporate both responsive and reference emitters within a single framework for internally calibrated detection.^113^ Dual-emissive UiO-derived frameworks coupling an oxygen-sensitive porphyrin with an oxygen-insensitive dye enable ratiometric O_2_ readout through triplet-state quenching;^113^ Cu/Tb co-doped Zn-MOF systems exploit competitive coordination to modulate energy transfer and restore Tb^3+^ emission;^114^ and peptide-linked fluorophores quenched by MOF surface proximity recover emission upon enzymatic cleavage, reporting proteolytic activity.^115^

Translation to biological environments is facilitated by selection of low-toxicity metal nodes (Zn^2+^, Fe^3+^, Zr^4+^) and biocompatible linkers such as terephthalic acid.^116,117^ However, long-term hydrolytic stability and metal ion leaching remain critical constraints that currently limit MOF-based sensing to shorter-duration or *ex vivo* applications.^116,117^

#### Silica matrices

Sol–gel condensation of alkoxide precursors such as tetraethyl orthosilicate (TEOS) or methyltriethoxysilane (MTES) yields siloxane networks whose porosity, hydrophilicity, and stiffness are tunable through precursor composition and hydrolysis ratio.^118,119^ Enzyme entrapment exploits the progressively tightening pore structure to simultaneously confine recognition elements, regulate analyte transport, and control the local solvation microenvironment.^118,120^ Pore microenvironment composition critically determines catalytic retention: rigid or hydrophobic matrices accelerate denaturation, whereas hydrophilic organosilane co-precursors preserve native activity.^121,122^ In gold nanoparticle-doped xerogel electrodes, silica precursor identity governs analyte partitioning, enzyme stability, and interfacial electrochemistry through a single compositional variable.^123^ Beyond enzymes, sol–gel silica accommodates antibodies, aptamers, DNAzymes, and living cells with enhanced long-term stability, because the siloxane cage restricts global biomolecule dynamics while permitting local motions required for analyte binding.^120,124^

The insulating silica backbone constrains electrochemical transduction to solution-phase mediator diffusion, rendering pore tortuosity and effective diffusion coefficients the primary determinants of both sensitivity and temporal response.^91^ Denser or more tortuous matrices improve selectivity by attenuating macromolecular interferent transport relative to small-molecule analytes, but simultaneously increase the time constant for analyte equilibration, creating a trade-off between selectivity and response speed that must be optimized for each monitoring application. Incorporating conductive additives circumvents this limitation: Xu *et al.* assembled reduced graphene oxide (rGO) onto Stöber-derived SiO_2_ nanospheres on a flexible carbon nanotube (CNT)–poly(dimethylsiloxane) (PDMS) base layer, achieving 60.8 µA mM^−1^ cm^−2^ sensitivity and a 3.7 µM limit of detection (LOD) in buffer, with 97–104% recovery in spiked sweat.^37^ Ordered mesoporous architectures (MCM-41,^125^ SBA-15^126^) mitigate transport limitations more fundamentally through uniform nanochannels that permit high-density enzyme immobilization while ensuring rapid analyte diffusion.^127^

Vertically ordered mesoporous silica nanochannel films (VMSF), prepared by electrochemically assisted self-assembly,^128^ extend this principle to charge-selective metabolite detection.^129,130^ The negatively charged silanol interior enriches cationic analytes such as dopamine (0.88 nM in buffer)^131^ while excluding macromolecular interferents; amine or carboxyl functionalisation inverts selectivity toward anionic metabolites such as urate (129 nM in buffer, with direct detection in 50-fold diluted whole blood).^130,132,133^ Integration with 3D macroscopic graphene achieves 23 nM uric acid detection,^134^ while glucose dehydrogenase immobilized on VMSF-modified indium tin oxide (ITO) enables electrochemiluminescent glucose detection (1.5 nM) through NADH-mediated enhancement of Ru(bpy)_3_^2+^ emission, with the luminophore enriched electrostatically in the nanochannels.^135^

In optical configurations, sol–gel silica coatings on optical fibers immobilize oxygen-sensitive luminophores while matrix permeability governs O_2_ diffusion and collisional quenching efficiency, with the sol–gel layer simultaneously serving as enzyme host, luminophore support, and diffusion-regulating membrane.^91,120^

#### Metal matrices

Metallic materials function as sensing matrices through electronic conductivity, plasmonic optical properties, and catalytic surface chemistry, with gold, platinum, and silver most extensively employed.^136,137^

In electrochemical sensing, the metal surface serves as the charge-transfer interface, the immobilization scaffold for biorecognition elements, and a principal determinant of electrical double layer structure and capacitance at the electrode–electrolyte interface.^138^ Electron-transfer kinetics depend on the electronic density of states, surface crystallographic orientation, and adsorbate layer composition.^139^ Platinum exploits intrinsic electrocatalytic activity toward H_2_O_2_ oxidation to directly transduce oxidase-catalyzed metabolite reactions without external mediators.^47^ Gold enables precise interfacial engineering through thiolate self-assembled monolayers (SAMs), where Au–S chemistry controls recognition element orientation, electron-transfer distance, and fouling resistance.^95^ Dervisevic *et al.* demonstrated this integration in a transdermal glucose monitoring patch (Si microneedle array/Au/ferrocene-poly(amidoamine)/GOx), where gold functioned simultaneously as conductive layer, immobilization scaffold, and electron transfer interface.^140^

Beyond biofouling, metallic electrodes face metabolite-level selectivity challenges intrinsic to their broad electrocatalytic activity. At the operating potentials required for H_2_O_2_ oxidation on platinum (∼+0.6 V *vs.* Ag/AgCl), endogenous electroactive species including ascorbic acid, uric acid, and acetaminophen undergo concurrent oxidation, generating faradaic currents indistinguishable from the target analyte signal.^49^ Permselective membranes (*e.g.*, Nafion, cellulose acetate) or low-potential mediator architectures mitigate this interference but introduce additional diffusion barriers or system complexity.^47^ This interferent susceptibility is a direct consequence of the same catalytic surface reactivity that enables sensitive detection, illustrating the design tension identified in the introduction.

Nanostructured gold and silver introduce localized surface plasmon resonance (LSPR) as an intrinsic transduction mechanism, with resonance frequency determined by particle geometry, interparticle spacing, and local dielectric environment.^141,142^ SERS configurations exploit electromagnetic hotspot confinement to amplify Raman cross-sections by several orders of magnitude.^136,143^ Tan *et al.* addressed the geometric constraint of hotspot reproducibility by assembling core–satellite plasmonic nanostructures *via* DNA tetrahedral scaffolds, enabling controlled hotspot formation and dual-modality *in vivo* microRNA-21 detection.^144^ Although most plasmonic metabolite sensors to date target glucose or uric acid *via* functionalised SERS base layers, the sensitivity of LSPR peak position to sub-monolayer refractive index changes makes the platform inherently suited to label-free small-molecule detection in low-volume biofluids.^145^

Biofouling constitutes the central limitation: nonspecific protein and lipid adsorption attenuates analyte access and alters interfacial impedance or refractive index.^146^ Antifouling strategies (PEG grafting, zwitterionic SAMs, polymer brushes) mitigate adsorption but reduce analyte flux or near-field accessibility.^147^ Composite architectures therefore integrate metallic nanostructures with porous supports, antifouling overlayers, or structured biological scaffolds to compensate for these intrinsic constraints.^47,145^ Despite these challenges, metallic matrices represent the most translationally advanced inorganic sensing platform: platinum-based enzyme electrodes form the transduction core of several commercial continuous glucose monitors, where composite membrane stacks address selectivity, biofouling, and calibration drift over 10.5–15.5 day operational lifetimes,^47,148–150^ whereas other systems use mediator-based electrodes (Section 3.1).

#### Metal oxide matrices

Metal oxides derive sensing functionality from ionic crystalline frameworks, semiconductor band structure, high surface hydroxyl density, and intrinsic electrocatalytic activity. ZnO, TiO_2_, CeO_2_, Fe_3_O_4_/Fe_2_O_3_, and CuO support electrochemical, optical, and magnetic modalities.^151^ According to the Gerischer model, charge transfer at semiconductor–electrolyte interfaces is governed by conduction band edge alignment relative to the solution redox level,^152^ determining the feasibility of direct electron transfer. TiO_2_ and ZnO isoelectric points (pH ∼ 6 and ∼9.5, respectively)^153,154^ provide pH-dependent surface charges exploitable for electrostatic enzyme immobilization that preserves active-site accessibility,^155,156^ while hierarchical morphologies further increase loading density and catalytic current.^157^

Non-enzymatic sensing leverages intrinsic oxide electrocatalysis toward direct metabolite oxidation.^158^ CuO operates through surface-confined Cu^3+^ species^159^ and NiO *via* the Ni^2+^/Ni^3+^ redox couple;^160^ AuNP-decorated CuO microelectrodes and AuNP-dispersed NiO nanodendrite arrays achieve sensitive glucose and lactate detection in serum and urine.^161,162^ Both systems, however, require alkaline media incompatible with physiological analysis, and competitive chloride adsorption can poison active sites.^158^ Beyond the pH constraint, non-enzymatic oxide electrodes exhibit limited discrimination among structurally similar metabolites: ascorbic acid, dopamine, and uric acid undergo oxidation at overlapping potentials on most oxide surfaces, and the absence of enzyme-mediated specificity renders the response susceptible to interference from any electroactive species present in the sample matrix.^151,158^ Certain oxides exhibit intrinsic nanozyme activity. In CeO_2_, reversible Ce^3+^/Ce^4+^ cycling coupled to oxygen vacancy dynamics produces multienzyme-mimetic activity, including peroxidase, oxidase, catalase, and superoxide dismutase functions, with efficiency scaling with vacancy density.^163,164^ This concept, broadened by the discovery of peroxidase-like Fe_3_O_4_ nanoparticles^165^ and generalized across oxide systems,^166^ has been applied to multiplexed colorimetric sugar detection through CeO_2_/Fe-MOF catalytic cascades.^38^ In CeO_2_-based systems, oxygen vacancy density, tunable through synthesis atmosphere and dopant incorporation, remains the primary structural variable governing turnover rate and analyte affinity.^164,167^ Fe_3_O_4_ further combines surface catalysis with superparamagnetic behaviour for field-directed assembly and analyte enrichment;^165,168,169^ Dagar *et al.* leveraged this in a composite lactate biosensor (Au/chitosan–Fe_3_O_4_–c-multiwalled CNT (c-MWCNT)/LOx) where Fe_3_O_4_ contributed enzyme anchoring and catalytic enhancement while carbon nanotubes compensated for oxide conductivity limitations.^170^ Oxide surface chemistry is highly sensitive to biological media composition: phosphate ions, amino acids, and serum proteins competitively adsorb, modifying surface charge,^171,172^ blocking catalytic sites, and altering analyte partitioning.^173^ Protective overlayers mitigate these effects but introduce diffusion barriers.^169^ Relative to metallic electrodes, metal oxide sensing platforms remain at an earlier translational stage: enzymatic oxide configurations have not yet demonstrated long-term calibration stability in undiluted biofluids comparable to commercial platinum-based glucose sensors.^47,151^

Across the four inorganic sub-classes, sensing behaviour emerges from composite interfacial architecture rather than isolated material properties.^91,98,136,166^ Each class is simultaneously enabled and constrained by its electronic structure and surface chemistry: plasmonic near-fields decay within ∼5–20 nm;^141,174^ oxide biofouling reflects high inorganic surface energies, which for metals reach 0.4–4 J m^−2^ compared with <0.05 J m^−2^ for organic polymers;^172,175^ the electronically insulating character of silica necessitates conductive additives;^91,176^ and MOF hydrolytic instability limits long-term order at physiological pH.^93^ These constraints reflect bond energies, band gaps, and interfacial thermodynamics,^175^ not engineering shortcomings amenable to incremental optimization. Translational maturity varies accordingly: among inorganic matrices, only metallic matrices have reached commercial deployment as the transduction core of continuous glucose monitors; silica requires integration with conductive materials for electrochemical transduction; and MOF- and oxide-based metabolite sensors remain at the proof-of-concept stage. For glucose as a benchmark analyte, this hierarchy is quantitative: metallic Pt electrodes in commercial CGMs quantify glucose across the clinically relevant millimolar range over the full sensor wear period,^150^ enzymatic VMSF–electrochemiluminescence (ECL) sensors reach nanomolar LODs but lack equivalent durability data,^135^ and non-enzymatic CuO/NiO systems achieve micromolar LODs only under non-physiological alkaline conditions.^161^ Whereas enzymatic sensors degrade through well-characterized inactivation kinetics,^177^ composite inorganic matrices fail through several coupled processes, including corrosion, dissolution, phase transformation, and biofouling, whose interactions make operational lifetime difficult to predict under physiological conditions.^47,178^

### 3.3 Carbon nanostructured matrices

Carbon adopts multiple hybridization states, giving rise to diverse structures with tunable physicochemical properties.^179^ In sensing systems, carbon-based materials function as electronically delocalized matrices in which signal propagation is governed by percolative charge transport, surface adsorption, and defect-mediated electronic modulation. These properties directly influence interfacial interactions, biomolecule immobilization, and charge-transfer efficiency. When structured at reduced length scales, carbon materials provide high accessible surface area, enhanced interfacial reactivity, and tunable electronic characteristics.^179^ Accordingly, carbon nanostructures have been widely explored in biosensing and biomedical applications due to their favorable physicochemical properties and amenability to functionalisation.^8,179,180^ While zero-dimensional carbon nanomaterials such as carbon quantum dots and fullerenes are frequently employed as optical signal-generating elements,^8^ they typically become matrix-relevant only when embedded within host matrices, where they can influence local photophysics, analyte partitioning, or interfacial signal coupling. The present discussion therefore focuses on higher-dimensional carbon architectures that more directly define interfacial structure and transport behaviour in sensing systems.

#### One-dimensional carbon nanostructures: carbon nanotubes

One-dimensional carbon nanostructures, including CNTs, carbon nanofibers, and carbon nanohorns,^181^ consist of extended sp^2^-conjugated frameworks that provide continuous conductive pathways along their longitudinal axis. Among these, CNTs are the most extensively investigated and are available in single- and multi-walled configurations.^182,183^ Owing to their delocalized π–electron network, CNTs form interconnected, high-surface-area conductive frameworks that serve as matrix layers in sensing architectures. Their electrical conductivity and structural stability support implementation in electrochemical and electronic platforms.^184–186^ In these systems, CNT networks are commonly incorporated as coatings or composite components to increase effective electroactive area and facilitate electron-transfer kinetics.^187,188^ Surface functionalisation further enables covalent or noncovalent immobilization of enzymes, antibodies, and other biorecognition elements while maintaining electrical connectivity.^189^ Importantly, sensing performance in CNT-based matrices is governed not solely by intrinsic nanotube conductivity but by network-level transport characteristics. In percolative films, charge transport depends on inter-tube junction resistance, alignment, and dispersion homogeneity, which collectively determine the effective conductivity of the matrix.^190^ Defect density and surface oxidation further modulate electronic structure and influence both catalytic behaviour and biomolecule attachment. Consequently, the analytical performance of CNT-integrated platforms reflects the combined optimization of electronic percolation, interfacial chemistry, and mass transport rather than a single material parameter.

#### Two-dimensional carbon nanostructures: graphene and derivatives

Two-dimensional carbon nanostructures consist of atomically thin sp^2^-bonded lattices in which charge transport occurs predominantly in-plane. Graphene and its derivatives are extensively investigated in sensing applications due to their high carrier mobility, large accessible surface area, and electrochemical stability.^8,184,191^ The fully exposed atomic surface renders electrical conductivity highly sensitive to adsorption-induced charge perturbations. This surface-dominated transport underlies graphenes function as an electronically active matrix. In field-effect configurations, interfacial binding events modulate channel carrier density and conductance.^192^ In electrochemical platforms, graphene supports rapid charge-transfer kinetics and high-density catalyst or biomolecule immobilization.^8,183,193^ Performance is governed by structural parameters including layer number, defect density, and heteroatom doping, which modify conductivity, catalytic activity, and interfacial charge-transfer behaviour.^183,184^ Restacking through π–π interactions reduces accessible surface area and restricts mass transport.^8,183^ Accordingly, the same surface accessibility that provides graphene with exceptional transduction sensitivity also renders it vulnerable to non-specific adsorption, necessitating composite or functionalised architectures for operation in complex media.^183,191^ As a representative example, Zambrano *et al.* described a strategy to render the surface of a carbon electrode fouling resistant. A bare laser-scribed graphene electrode exhibited a retention of only 53.7% of its initial electrochemical response following 24 h exposure to bovine serum albumin protein. The application of the antifouling layer provided instead a retention of 91.5% following the same 24 h exposure. Microscopic and electrochemical characterization attributed this loss to nonspecific protein adsorption that blocked the porous graphene surface and increased charge-transfer resistance.^194^

#### Three-dimensional carbon nanostructures

Three-dimensional carbon nanostructures comprise interconnected porous frameworks assembled from nanoscale building blocks such as graphene sheets, carbon fibers, or carbon nanotubes, forming aerogels, foams, or sponge-like architectures.^195,196^ Unlike two-dimensional sheets or one-dimensional networks, these structures introduce hierarchical porosity and volumetric conductivity within a continuous carbon matrix. The combination of high surface area and interconnected conductive pathways enables simultaneous mass transport and charge propagation throughout the framework, rendering 3D carbon architectures effective matrix-forming components in electrochemical and electronic sensing systems.^197,198^ The porous network facilitates rapid analyte diffusion while maintaining electrical contact with immobilized catalysts or biorecognition elements, thereby integrating transport and transduction within a single structural body.^197–199^ Analytical performance is governed by pore size distribution, network connectivity, and structural density, which collectively determine diffusion resistance, electroactive surface accessibility, and effective conductivity. However, mechanical fragility, structural collapse upon drying, and limited control over dispersion can constrain practical implementation. Consequently, 3D carbon matrices uniquely integrate volumetric mass transport with continuous electronic conductivity, but require hybridization with polymers, metals, or oxides to achieve the mechanical and interfacial stability needed for sustained operation.^197,199^

#### Cross-modality roles and system-level integration

Carbon nanostructured matrices function across electrochemical, field-effect, and optical platforms through a common principle: network architecture, defect density, and surface functionalisation collectively govern performance rather than intrinsic conductivity alone.^192,199–201^ Carbon–carbon composites such as graphene–CNT architectures further reduce interfacial resistance while preserving surface area,^202,203^ and carbon nanostructures additionally provide mechanical reinforcement within composite interfaces.^204,205^ However, batch-to-batch variability in defect density and surface chemistry, combined with susceptibility to non-specific adsorption, mean that carbon matrices primarily function as electron-transfer facilitators within composite architectures rather than as standalone sensing platforms.^8,184^

### 3.4 Supramolecular matrices

In this review we focus on those supramolecular matrices that are based on host–guest interactions between a macrocyclic host and a smaller guest molecule.^206^ In classical host–guest sensing interfaces, these noncovalent interactions enable reversible binding of the target analyte within a defined cavity, thereby introducing molecular recognition directly into the matrix layer. Unlike enzymatic sensors, where signal generation depends on catalytic turnover and enzyme kinetics,^207^ or diffusion-limiting membranes that regulate analyte flux primarily through pore size, tortuosity, and diffusion coefficients,^208^ supramolecular matrices concentrate analytes at the interface through equilibrium complex formation. The surface density of the analyte is governed by the association constant (*K*_a_), which depends on cavity geometry and functionality, hydrophobic desolvation, and electrostatic complementarity.^209,210^ For cucurbiturils and related macrocycles, reported *K*_a_ values can span several orders of magnitude, enabling substantial interfacial encapsulation of guest molecules.^211^ Analytical performance is therefore coupled to binding thermodynamics and exchange kinetics. Host–guest affinity determines equilibrium enrichment at the interface and can enhance signal amplitude, whereas exchange kinetics, particularly the dissociation rate, determine response and recovery times. High-affinity systems may still exhibit rapid exchange, while weaker-binding systems can equilibrate rapidly but provide lower interfacial analyte enrichment. The sensor response thus reflects the interplay between association/dissociation rates and the specific transduction mechanism employed. This equilibrium-controlled recognition is implemented in sensing matrices primarily through a small number of structurally defined macrocyclic host families. Macrocyclic hosts used in metabolite sensing include cyclodextrins (CDs), cucurbiturils, calixarenes, and pillararenes.^210,211^ Cyclodextrins consist of *α*-1,4-linked glucose subunits forming truncated cone-shaped structures with hydrophobic inner cavities and hydrophilic rims. Cucurbiturils are composed of glycoluril units bridged by methylene groups and present carbonyl-lined cavities that favor binding of cationic or electron-deficient guests. Calixarenes and pillararenes are constructed from phenolic and bisphenolic subunits, respectively; their aromatic cavities support π–π, hydrophobic, and electrostatic interactions depending on functionalisation. The cavity size and functionality, and electronic environment determine binding affinity, selectivity profiles, and exchange kinetics in aqueous media.

#### Scaffolding monolayers

In multilayer sensing architectures, macrocycles can function as supramolecular linkers that couple conductive layers to those for biomolecular recognition. Typically, the underlying electrode or electropolymerized film is covalently modified with the macrocyclic host, while the outer recognition layer carries a complementary guest functionality. Host–guest complexation then enables reversible and spatially controlled immobilization of the bioactive scaffold, separating biomolecular anchoring from direct covalent attachment to the electrode surface and permitting controlled surface density and multivalent presentation of recognition elements.^42^ A representative implementation employed an electropolymerized pyrrole–carboxylic acid film on gold, subsequently functionalised with *β*-cyclodextrin units to provide encapsulation sites for an adamantane-modified gliadin.^42^ The resulting supramolecular interface (Au/PPy/CD/ADA–CMC–GLI; ADA, adamantane; CMC, carboxymethylcellulose; GLI, gliadin) enabled amperometric detection of anti-gliadin antibodies. Comparative surfaces lacking either the cyclodextrin layer or the conductive polypyrrole scaffold exhibited markedly reduced signal responses, demonstrating that analytical performance arises from the synergistic integration of supramolecular organization and electronic conductivity. These findings indicate that macrocycles in immobilization architectures regulate interfacial spacing, orientation, and local recognition-element density, thereby influencing electron-transfer and effective signal amplification. Consequently, analytical sensitivity depends on matrix architecture and interfacial organization rather than solely on the intrinsic affinity or activity of the recognition element.

#### Porous materials

Supramolecular macrocycles regulate analyte distribution at the sensing interface through reversible encapsulation complex formation. Unlike diffusion-limited membranes, where transport is primarily dictated by pore size and tortuosity, host–guest matrices introduce thermodynamically defined docking domains. The resulting encapsulation equilibrium increases the local analyte concentration at the transducer surface and can enhance signal amplitude without relying on catalytic turnover. Embedding cyclodextrins into porous polymer frameworks creates distributed encapsulation sites throughout the matrix interface. Porous *β*-cyclodextrin polymers based on tetrafluoroterephthalonitrile networks exhibit markedly higher uptake compared to non-porous cyclodextrin materials, reflecting cooperative benefits between accessible porosity and host–guest binding.^212^ In electrochemical systems, selective encapsulation has been combined with nanostructured conductive layers. Thiolated *β*-cyclodextrin assembled on nanoporous gold couples selective encapsulation with high interfacial area and efficient charge transfer.^44^ In such architectures, analytical performance is governed by the interplay between binding thermodynamics, mass transport within the porous network, and electron-transfer kinetics at the electrode interface.

#### Nanostructures

Supramolecular macrocycles are rarely implemented as standalone sensing layers; instead, they are integrated into composite architectures that couple affinity-driven recognition with catalytic, plasmonic, or conductive scaffolds. In these systems, the macrocycle governs analyte localization and selectivity, while the supporting nanostructure determines signal transduction efficiency. Cyclodextrins and cucurbiturils have been employed as stabilizing and structure-directing agents during metal nanoparticle synthesis, where the macrocycle-to-metal ratio influences nanoparticle size and morphology.^213^ Surface-bound macrocycles provide accessible encapsulation sites that position analytes within plasmonic hot spots, thereby enhancing SERS signals.^43,214,215^ In such configurations, supramolecular recognition controls interparticle spacing and guest positioning, whereas electromagnetic amplification arises from the metallic nanostructure. Integration with graphene and related carbon nanomaterials further illustrates the complementary roles of affinity and conductivity. The combination of macrocycles with graphene derivatives has been recognized as a promising strategy to merge host–guest selectivity with high charge-carrier mobility and large specific surface area.^216^ Functionalisation of rGO with *β*-cyclodextrin enhances electrochemical sensitivity by coupling encapsulation-mediated affinity with efficient electron transfer.^217^ Similarly, calixarene- and pillararene-modified graphene platforms translate competitive guest exchange into fluorescence modulation through energy-transfer mechanisms.^218,219^ More complex hybrid systems incorporate macrocycles into porous polymers, covalent organic frameworks, or metal-ion-containing composites to co-localize binding sites and catalytic centers within a single matrix.^220,221^ In these architectures, supramolecular recognition defines selectivity, while the composite scaffold governs conductivity, catalytic activity, or optical amplification.

#### Translational constraints and selectivity limitations

Despite their structural versatility, supramolecular matrices face intrinsic challenges in complex biological media. Host–guest interactions are typically governed by size, shape, and electrostatic complementarity rather than strict molecular specificity. As a result, structurally related metabolites or competing biomolecules may occupy the macrocyclic cavity, reducing effective selectivity under physiological conditions.^42^ Moreover, because signal generation relies on equilibrium encapsulation rather than catalytic turnover, LODs are fundamentally constrained by the association constant (*K*_a_) and the sensitivity of the transduction mechanism. High association constants enhance interfacial analyte enrichment, but response kinetics are governed by association and dissociation rates rather than by affinity alone. Continuous monitoring therefore requires host–guest systems that combine sufficient equilibrium enrichment with rapid and reversible exchange.

It should be noted that, relative to polymeric and carbon-based matrices, supramolecular sensing platforms for metabolite detection remain at an earlier stage of translational development. While host–guest recognition has been demonstrated in buffered solutions and, in selected cases, in diluted biological samples, long-term continuous operation in undiluted physiological fluids or *in vivo* has not yet been reported for macrocycle-based metabolite sensors. The absence of operational stability data under chronic implantation conditions represents a significant gap that must be addressed before supramolecular matrices can be considered viable candidates for continuous metabolite monitoring applications. Bridging this gap will likely require integration of macrocyclic recognition elements into composite architectures that provide the mechanical compliance, antifouling resistance, and calibration stability demonstrated by more mature polymeric platforms.

## 4 Comparative analysis: matrix–mechanism–application coupling

The preceding sections demonstrate that matrix materials are not passive supports but active determinants of signal generation, analyte partitioning, and long-term operational stability. A comparative analysis across material classes shows that differences in analytical performance arise from how each matrix architecture modulates three fundamental parameters: (i) analyte transport, (ii) interfacial organization, and (iii) signal transduction efficiency. Rather than categorizing matrices by composition alone, it is therefore instructive to compare them through a matrix–mechanism–application framework.

### 4.1 Mechanistic control of signal generation

Polymeric matrices primarily regulate diffusive transport and spatial immobilization of recognition elements. Crosslink density, porosity, and hydration state determine analyte flux toward embedded enzymes or redox centers. Signal intensity in such systems is therefore frequently transport-limited, particularly under continuous monitoring conditions. In contrast, supramolecular matrices introduce thermodynamically defined partitioning domains governed by host–guest association constants (*K*_a_). Here, local analyte concentration at the transducer interface is controlled by equilibrium binding rather than solely by diffusional permeability. Signal modulation arises from affinity-driven enrichment and reversible exchange kinetics. Carbon-based matrices and conductive polymers emphasize electron-transfer facilitation. Their high surface area and charge-carrier mobility reduce interfacial resistance and enable rapid signal propagation, often shifting the rate-limiting step away from charge transfer and toward enzymatic turnover or mass transport. Inorganic nanostructures and metal-based composites frequently contribute catalytic or plasmonic amplification. In these systems, matrix activity directly enhances signal magnitude through electrocatalytic reactions or localized electromagnetic field enhancement, rather than through transport or affinity control alone. These distinctions highlight that analytical sensitivity does not depend solely on intrinsic material properties, but on how the matrix architecture defines the dominant signal-generating mechanism. Dynamic performance is governed by a different rate-limiting process in each matrix class (Table 2). In polymeric and hydrogel matrices, response and recovery times are transport-limited and scale with the characteristic diffusion time *L*^2^/*D*, where *L* is the layer thickness and *D* the effective diffusion coefficient; halving the layer thickness therefore reduces the response time approximately four-fold, whereas increasing crosslink density prolongs it through a lower *D* (Section 3.1). In supramolecular matrices, recovery is limited by the dissociation rate of the host–guest complex, and in stimuli-responsive hydrogels by bulk network rearrangement, which additionally causes hysteresis. In carbon-based and inorganic matrices, dynamic behaviour is determined by pore diffusion in frameworks such as MOFs and by fouling that progressively blocks the accessible surface. Consequently, response time is optimized through layer thickness and mesh size in polymeric matrices, through dissociation kinetics in supramolecular matrices, and through pore architecture and antifouling design in carbon-based and inorganic matrices. To contextualize these mechanistic relationships across representative experimental implementations, the corresponding literature is summarized in Table 2.

**Table 2:** Matrix–mechanism–application comparison of major matrix classes with representative quantitative performance indicators. Values represent ranges reported across cited studies and are intended as approximate benchmarks; performance is strongly dependent on specific matrix composition, transduction mechanism, and operating conditions. Symbols: EC = electrochemical, OPT = optical, EL = electrical

Matrix class	Dominant mechanism	Signal coupling	Response time	Operational stability	Key strengths	Primary limitations
Polymeric matrices	Diffusion control; enzyme immobilization	Transport-limited EC or OPT response	Seconds to minutes (thickness- and crosslink-dependent)	Hours to one year (up to 15.5 d in transcutaneous CGM; 365 d in an implanted fluorescence-based CGM)	Biocomp. mechanical tunability; enzyme compatibility	Swelling-induced drift; biofouling; dehydration
Supramolecular matrices	Equilibrium enrichment (*K*_a_-controlled)	Affinity-modulated EC or OPT response	Seconds to minutes (*K*_a_- and exchange-rate-dependent)	Limited long-term data; hours demonstrated *in vitro*	Tunable selectivity; reversible binding; modular design	Competitive binding; limited molecular specificity; scarce *in vivo* validation
Carbon-based matrices	Electron-transfer facilitation	Low-resistance charge transfer (EC, FET)	Sub-second to seconds (fast e^−^ transfer kinetics)	Days to weeks (fouling-dependent)	High conductivity; fast response; mechanical flexibility; large surface area	Non-specific adsorption; surface fouling; batch-to-batch variability
Inorganic/nanostructured matrices	Catalytic or plasmonic amplification	Electrocatalytic or SERS enhancement	Seconds (oxides/metals) to minutes (MOF diffusion-limited)	Hours to days (limited in physiological fluids)	High sensitivity; signal amplification; thermal/chemical stability	Corrosion; limited biocompatibility; mechanical mismatch with tissue

### 4.2 Same-recognition-element comparisons across matrices

The mechanistic role of the matrix is most directly demonstrated when the recognition element and transduction principle are held constant while the matrix is varied. Four systems illustrate this. For oxygen sensing, Pt(ii)–pivaloyl porphyrin embedded as the identical indicator in TEOS sol–gel, PEMA, the Cavilon terpolymer, and PPMA yields photobleaching rates spanning more than an order of magnitude (5.0, 2.9, 1.8, and 0.2% h^−1^, respectively), humidity sensitivities ranging from a dry-to-humid ratio of 2.1 in pure TEOS sol–gel to insensitivity in the acrylates, and Stern–Volmer parameters *k* = 0.044, 0.059, 0.073, and 0.074 mmHg^−1^ for PEMA, PPMA, TEOS, and Cavilon, respectively.^66,67^ Photobleaching does not follow oxygen accessibility, which the Stern–Volmer constant integrates through oxygen solubility and diffusivity in the matrix:^22,51^ TEOS and Cavilon exhibit nearly identical constants but differ 2.8-fold in photobleaching rate, and PPMA combines a higher constant than PEMA with a 15-fold lower photobleaching rate. Photostability is therefore governed by matrix-specific interactions with the dye rather than by oxygen transport alone. In PDMS, a biexponential phosphorescence decay indicates aggregation of the same porphyrin, to which the authors attributed self-quenching, singlet-oxygen photodegradation, and lower photostability than in PEMA and PPMA.^66^ For glucose sensing, GOx with H_2_O_2_ amperometry reaches 0.184 µA mM^−1^ sensitivity, dynamic range up to 22 mM, and 23 µM LOD in gold-nanoparticle-doped (3-mercaptopropyl)trimethoxysilane (MPTMS) xerogels in buffer, compared with 0.072 µA mM^−1^ and 12 mM without doping,^123^ 10.5–15.5 day *in vivo* lifetime with 8.0–9.1% mean absolute relative difference (MARD) in cross-linked polyurethane- or PEG-based commercial CGM matrices,^149,150^ and 60.8 µA mM^−1^ cm^−2^ with a 3.7 µM LOD in rGO-on-silica composites on CNT–PDMS layers in buffer;^37^ these figures of merit are not directly comparable because the matrix shifts which step limits the response (analyte flux, H_2_O_2_ confinement, enzyme stability, or biocompatibility) and which operating environment is targeted. For host–guest sensing, *β*-cyclodextrin with an adamantane-anchored gliadin construct detecting anti-gliadin antibody yields amperometric sensitivities of 0.45, 0.13, 0.08, and 0.05 µA mL mg^−1^ and limits of detection of 33, 135, 240, and 250 ng mL^−1^ on Au/PPy/CD/ADA–CMC–GLI, Au/PPy/GLI, Au/CD/ADA–CMC–GLI, and Au/C3SAM/GLI, respectively, despite comparable gliadin surface coverage across the four surfaces.^42^ Although the analyte is a protein rather than a metabolite, this system is included because it isolates the matrix contribution under otherwise identical recognition and transduction conditions. A nine-fold sensitivity enhancement and 7.6-fold LOD improvement therefore arise from matrix architecture alone. VMSF-based dopamine sensors further demonstrate matrix-dependent performance. For differential pulse voltammetry (DPV) in phosphate buffer (pH 6.0), VMSF grown on (3-aminopropyl)triethoxysilane (APTES)-grafted glassy carbon electrodes (GCE) and on electrochemically pre-activated GCE (p-GCE) yield LODs of 7.8 and 0.88 nM, respectively,^131,222^ showing how the underlying conductive layer alters electron-transfer efficiency and dopamine enrichment within the silica nanochannels. In 50-fold diluted human serum, VMSF/p-GCE retained 97% of its buffer signal, compared with approximately 53% for the uncoated p-GCE.^131^ Ru(bpy)3^2+^ Across all four systems, the matrix determines which mechanism limits performance and which figure of merit is optimized; quantitative figures of merit for these comparisons are summarized in Table 3.

**Table 3:** Cross-matrix comparisons holding the recognition element and transduction principle constant. Quantitative values are reported as in the primary sources cited

System (recognition/transduction)	Matrix variant	Reported figure of merit	Matrix function isolated	Ref.
Pt(ii)–pivaloyl porphyrin/phosphorescence quenching by O_2_	TEOS sol–gel	Photobleaching 5.0% h^−1^;	Transport (O_2_ diffusivity); microenvironment (water uptake); photostability	66 and 67
		humidity ratio 2.1;		
		*k* = 0.073 mmHg^−1^		
	Cavilon terpolymer	Photobleaching 1.8% h^−1^;		
		humidity-insensitive;		
		*k* = 0.074 mmHg^−1^		
	PEMA	Photobleaching 2.9% h^−1^;		
		humidity-insensitive;		
		*k* = 0.044 mmHg^−1^		
	PPMA	Photobleaching 0.2% h^−1^;		
		humidity-insensitive;		
		*k* = 0.059 mmHg^−1^		
GOx/H_2_O_2_ amperometry	MPTMS xerogel + PU cap (undoped)	Sensitivity 0.072 µA mM^−1^;	Enzyme immobilization; H_2_O_2_ confinement	123
		dynamic range up to 12 mM (buffer)		
	Au-nanoparticle-doped MPTMS xerogel + PU cap	Sensitivity 0.184 µA mM^−1^;	Enzyme immobilization; H_2_O_2_ confinement; interferent exclusion; electron-transfer extension into the film	123
		dynamic range up to 22 mM;		
		LOD 23 µM (buffer)		
	Cross-linked PU/PEG (commercial CGM)	10.5–15.5 d *in vivo* lifetime;	Biocompatibility; long-term diffusion balance	149 and 150
		MARD 8.0–9.1%		
		(subcutaneous ISF)		
	rGO/SiO_2_ on CNT-PDMS	Sensitivity	Conductivity enhancement; flexible support	37
		60.8 µA mM^−1^ cm^−2^;		
		LOD 3.7 µM (buffer)		
*β*-cyclodextrin/ADA-anchored gliadin antibody amperometry	Au/PPy/CD/ADA–CMC–GLI	Sensitivity 0.45 µA mL µg^−1^;	Synergistic immobilization scaffold + conductive support	42
		LOD 33 ng mL^−1^ (buffer)		
	Au/PPy/GLI (no CD)	Sensitivity 0.13 µA mL µg^−1^;	Conductive support only	
		LOD 135 ng mL^−1^ (buffer)		
	Au/CD/ADA–CMC–GLI (no PPy)	Sensitivity 0.08 µA mL µg^−1^;	Host–guest organization only	
		LOD 240 ng mL^−1^ (buffer)		
	Au/C3SAM/GLI (neither)	Sensitivity 0.05 µA mL µg^−1^;	Reference SAM	
		LOD 250 ng mL^−1^ (buffer)		
VMSF/Dopamine DPV	VMSF/APTES/GCE	Range: 10 nM–100 µM;	Enrichment; antifouling; selective transport	222
		LOD: 7.8 nM (buffer)		
	VMSF/p-GCE	Ranges: 10 nM–1 µM,	Improved adhesion; enrichment; fast electron transfer	131
		1–30 µM;		
		LOD: 0.88 nM (buffer)		

## 5 Translational potential and future directions

The translational requirements for matrix-based sensing are most concretely illustrated by CGM, the only matrix-engineered continuous metabolite sensor to achieve widespread clinical deployment. Transcutaneous electrochemical systems achieve operational lifetimes of 10.5–15.5 days with MARD of 7.8–9.1%,^149,150,223,224^ and an implanted fluorescence-based sensor reaches 365 days with a MARD of 8.8%.^225^ Critically, the dominant failure mode is not enzymatic degradation but matrix-level processes: membrane biofouling, fibrotic encapsulation, and progressive changes in effective diffusion coefficients that necessitate factory calibration algorithms.^48^ Biocompatibility coatings suppress protein adsorption and attenuate the foreign body response that would otherwise progressively isolate the sensing interface.^226,227^ CGM thus demonstrates that matrix engineering is a critical component of real-world continuous sensing, but only when integrated with appropriate transduction mechanisms, calibration strategies, and signal processing algorithms. Extending these principles to multi-metabolite platforms, longer operational lifetimes, and more complex biological environments defines the central translational challenge addressed in the following sections.

### 5.1 Matrix architectures for multi-metabolite sensing

Future sensing platforms will increasingly require simultaneous detection of multiple metabolites within a shared physiological environment. This demands matrix architectures capable of spatial and functional compartmentalization: orthogonal recognition domains must be integrated within a common scaffold while minimizing cross-reactivity, signal interference, and diffusion coupling between co-localized analytes. Reported magnitudes vary widely: in a dual glucose/lactate sensor, glucose-induced cross-talk at the lactate electrode was reported to be below 3% of the lactate response, whereas no detectable lactate cross-talk was observed at the glucose electrode.^228^ Interference from coexisting electroactive species can be considerably larger; in a needle-type glucose/lactate sensor with a poly(1,3-phenylenediamine) barrier, ascorbate, urate, and acetaminophen produced responses corresponding to 1.8%, 9%, and 23%, respectively, of the signal generated by 1 mM lactate,^229^ while in a multiplexed microneedle platform, coexisting ions produced approximately 2.7–14.1% interference depending on the sensing channel and interferent.^230^ Each matrix class approaches this challenge through distinct structural mechanisms. Polymeric matrices enable multilayer or gradient structures that spatially separate enzyme domains or redox mediators, but swelling-induced structural changes may alter diffusion pathways over extended operation. Supramolecular systems offer modular affinity sites tunable through host selection or functionalisation, enabling selective partitioning of chemically distinct analytes, though competitive binding and exchange kinetics complicate multiplexed equilibrium sensing unless orthogonal host systems are employed. Carbon-based and nanocomposite matrices provide high-density electrode integration and low-noise signal propagation, facilitating multiplex electrochemical arrays. Inorganic plasmonic architectures enable optical multiplexing through spectrally distinct enhancement domains, though long-term implantation stability remains a limiting factor. Across all classes, matrix selection should be driven not by material taxonomy but by identifying the dominant rate-limiting step and aligning matrix architecture with the intended sensing mechanism, clinical context, and the requirement to isolate analyte signals at the microscale.

### 5.2 Toward calibration-free operation

Long-term continuous sensing is limited not only by analyte selectivity but also by calibration drift arising from matrix evolution. Swelling, biofouling, enzyme degradation, and competitive binding alter effective diffusion coefficients, binding equilibria, and interfacial resistance over time. Signal-processing algorithms partly compensate for this drift. CGM calibration has evolved from linear regression against fingerstick reference values to signal-processing, modelling, and machine-learning approaches, including time-varying calibration functions that account for sensor drift.^231^ Factory calibration, which eliminates user calibration, depends directly on the matrix: the enzyme layer and the glucose-limiting membrane determine sensor sensitivity, and their reproducible deposition is a prerequisite for assigning a single calibration factor.^50^ In a pilot study, the sensor-to-sensor coefficient of variation of sensitivity increased from 4.7% *in vitro* to 8.3% *in vivo*, yet a single universal calibration factor yielded a MARD of 10.4%, comparable to 10.9% with fingerstick calibration.^232^ Algorithms therefore correct predictable sensitivity changes, whereas the foreign body response can alter the sensor response during wear and must be minimized through membrane chemistry and sensor design.^50^ Future matrix strategies must therefore stabilize the dominant signal-generating mechanism. Approaches include mechanically stable hydrogel networks with minimized volumetric drift, supramolecular systems engineered for controlled exchange kinetics, and composite architectures that incorporate internal referencing or redundant sensing pathways. Matrix designs that decouple analyte response from environmental perturbations will be essential for reducing recalibration frequency in wearable and implantable devices.

### 5.3 Advanced fabrication and sustainable materials

Emerging fabrication strategies offer new opportunities to engineer matrix architecture with high spatial precision. Additive manufacturing and 3D printing enable controlled porosity, layered functionality, and patient-specific geometries, facilitating integration of diffusion barriers, catalytic domains, and recognition layers within a single construct. In parallel, biodegradable and bioresorbable matrices are gaining relevance for transient sensing applications. Designing materials that maintain predictable transport and recognition properties during controlled degradation remains a significant materials challenge. Future work must link degradation kinetics to sensing performance to ensure reliable operation throughout the device lifetime.

### 5.4 Data-driven approaches to matrix design

The rational design of sensing matrices involves navigating a high-dimensional parameter space encompassing polymer composition, crosslink density, pore architecture, surface chemistry, and composite stoichiometry, where coupled trade-offs between transport, stability, and signal transduction make purely empirical optimization inefficient. Machine learning (ML) approaches are beginning to address this challenge at multiple levels of the sensing pipeline. At the materials level, ML models have been applied to predict structure–property relationships in polymer and nanocomposite systems, enabling accelerated screening of candidate matrix compositions for target permeability, mechanical compliance, or interfacial stability.^233^ As a representative example, Yang *et al.* developed an interpretable multitask ML framework to predict gas permeability directly from polymer structure, achieving test-set *R*^2^ values of approximately 0.9 and subsequently screening more than nine million hypothetical polymers. The approach identified candidate materials with tailored transport characteristics, illustrating how data-driven screening can substantially narrow the experimental search space for matrix compositions with desired permeability profiles.^234^ At the device level, ML-assisted signal processing has demonstrated the ability to compensate for matrix-related drift and fouling artifacts during continuous operation, effectively extending functional sensor lifetime beyond the intrinsic stability limits of the matrix itself.^235^ For instance, ML algorithms trained on time-dependent impedance or amperometric data can distinguish analyte-driven signals from baseline shifts caused by progressive membrane degradation or biofouling, reducing the frequency of recalibration.^236^ Heng *et al.*, for example, developed a semi-supervised adversarial domain-adaptation convolutional neural network (SAD-CNN) for electronic nose arrays that aligned drifted sensor-response distributions with the original calibration domain, achieving classification accuracies of 78.0–82.5% under long-term drift conditions while reducing dependence on newly labeled calibration data.^237^ In multiplexed platforms, pattern recognition algorithms can further deconvolve overlapping responses from cross-reactive matrix–analyte interactions, relaxing the stringent selectivity requirements imposed on individual sensing channels.^236^ While these approaches do not replace mechanistic matrix design, they offer a complementary strategy for navigating the complex interplay between matrix properties, operating environment, and analytical performance, and are likely to play an increasingly important role as sensing platforms scale toward multi-metabolite, continuous, and clinically deployed configurations.

### 5.5 Regulatory and clinical considerations

Translation of matrix-based sensors into clinical practice requires reproducible fabrication, long-term stability, and validated biocompatibility. Matrix materials must withstand sterilization processes, maintain structural integrity *in vivo*, and exhibit minimal leaching or inflammatory response. Regulatory approval will depend not only on sensing accuracy but also on the stability of matrix architecture under physiological stress. Establishing standardized testing protocols for drift, fouling resistance, and mechanical durability will therefore be critical for advancing matrix-driven sensing technologies toward clinical deployment.

## 6 Conclusions

The comparative analysis presented in this review establishes that metabolite sensor performance is governed not by transduction technology alone, but by the alignment between matrix architecture and the dominant signal-generating mechanism. Matrix selection is therefore a primary design parameter, not a secondary engineering choice. Polymeric matrices primarily govern diffusive transport; supramolecular matrices introduce equilibrium-controlled analyte enrichment; carbon-based matrices facilitate rapid electron transfer; inorganic and nanostructured matrices provide catalytic or plasmonic amplification. No single class simultaneously optimizes sensitivity, selectivity, and stability, and performance emerges from how well the matrix architecture addresses the physicochemical step that most constrains signal output in a given sensing context.

This dependence is clearest when recognition chemistry and transduction principle are held constant and only the matrix is varied, conditions under which matrix composition alone produces substantial differences in sensitivity and operational stability. Rational sensor design therefore begins by identifying the step that most constrains signal output, whether analyte diffusion, interfacial charge transfer, equilibrium binding, or catalytic turnover, and proceeds by selecting a matrix whose properties specifically address that constraint. As metabolite sensing advances toward continuous, multiplexed, and clinically deployable platforms, this mechanistic alignment will determine which systems sustain calibration-stable performance in complex biological environments.

## Author contributions

Martin Varga: conceptualization, investigation, writing – original draft, writing – review & editing. Guglielmo Risi: conceptualization, investigation, writing – original draft, writing – review & editing. Saiyyna Stepanova: investigation, writing – original draft, writing – review & editing. Mahsa Nasehi: investigation, writing – original draft, writing – review & editing. Celal Bahtiyar: investigation, writing – original draft, writing – review & editing. Emmanouil Rousakis: conceptualization, supervision, funding acquisition, investigation, writing – review & editing. Lilian Witthauer: conceptualization, supervision, funding acquisition, project administration, investigation, writing – original draft, writing – review & editing.

## Conflicts of interest

There are no conflicts to declare.

## Supplementary Material

TB-OLF-D6TB01338D-s001

## Data Availability

No primary research results, software or code have been included and no new data were generated or analysed as part of this review. Supplementary information (SI): Table S1 Matrix-enabled sensing platforms categorized by sensing modality, mechanism, and associated matrix function. See DOI: https://doi.org/10.1039/d6tb01338d.
