# Supplementary material for: Matrix materials as functional enablers in metabolite sensing: from mechanistic demands to translational potential
Source: J Mater Chem B. 2026 Oct 30. Online ahead of print. doi: 10.1039/d6tb01338d (PMC13729244; doi:10.1039/d6tb01338d)
Supplement: Supplementary file 1 [file TB-OLF-D6TB01338D-s001.pdf]

Supplementary Information (SI) for Journal of Materials Chemistry B.
This journal is © The Royal Society of Chemistry

Supplementary information

Table S1 Matrix-enabled sensing platforms categorized by sensing modality, mechanism, and associated matrix function

Mechanism	Representative Implementation	Matrix Function
**Optical**		
Luminescence	Calixarene functionalised on MnO_2@rGO [218]	Encapsulation; FRET-mediated quenching; selective analyte capture
	Pillararene-functionalised graphene oxide (CP6@rGO) [219]	Encapsulation; competitive host–guest exchange modulates emission
	Eu^{3+}-doped UiO-66 (EuUPDC) [112]	Selective quenching; host–guest interactions
	Eu(III)-MOF JXUST-55 for tryptophan and 5-HIAA [36]	Porous confinement; selective intracellular quenching
	PEGDA-co-PAAm hydrogel microneedle (FRET glucose) [57]	Hydrated host network; FRET pair immobilization; diffusion control
	Pt(II)-porphyrin in PPMA matrix on optical fiber [67]	Luminophore immobilization; O_2 diffusion control; photostable interface
	Dual-emissive UiO-derived frameworks (porphyrin/dye) [113]	Internal calibration; O_2-responsive triplet quenching
	Cu/Tb co-doped Zn-MOF [114]	Competitive coordination modulates energy transfer; ratiometric readout
	MOF with peptide-linked fluorophores [115]	Spatial confinement; proximity-dependent energy transfer; enzyme-cleavage readout
	Phosphorescent O_2 indicator in acrylate thin film [66]	Luminophore immobilization; controlled O_2 permeability; Stern–Volmer transduction
Colorimetry	Calixarene-capped Au nanoparticles [221]	Encapsulation; selective host–guest recognition drives colorimetric shift
	CeO_2 NPs@Fe-MOF cascade platform [38]	Peroxidase-like nanozyme activity; spatially organized cascade amplification
Raman / SERS	Surface-bound CDs on metal nanoparticles [213]	Macrocycle encapsulation positions analyte in plasmonic hotspot
	β-CD/α-Fe_2O_3@AgNP matrix [43]	CD-directed analyte capture; AgNP scaffold provides electromagnetic enhancement
	DNA-scaffolded AuNP core–satellite structures [144]	Hotspot control; geometric scaffolding
	Agarose hydrogel [24]	Immobilization; mechanical stability; sweat-compatible diffusion medium
Refractive index shifts	Polyglycerol–PEG hydrogel [53]	Anti-fouling; hydrated sensing interface
	PVA-coated flexible fiber optic sensor (acetone) [68]	Analyte-induced plasticization shifts cavity length; refractive-index modulation
	Graphene-assisted flexible SPR chip [41]	Evanescent field amplification; enhanced biomolecule adsorption
LSPR	Nanostructured Au/Ag matrices [141,142]	Near-field enhancement
**Electrochemical**		
Amperometric	Hydrogel microneedle array for ISF metabolite sampling [59]	ISF extraction; diffusion control; mechanical insertion strength
	PtNP-decorated polyaniline conductive hydrogel [27]	Enzyme immobilization; electron transfer; catalytic amplification
	Au-coated Si microneedle array (Fc-PAMAM/GOx) [140]	Conductive layer; enzyme immobilization; electron-transfer interface
	AuNP-decorated CuO microelectrodes [161]	Non-enzymatic electrocatalysis via surface Cu^{3+} species
	AuNP-dispersed NiO nanodendrite arrays [162]	Non-enzymatic electrocatalysis via Ni^{2+}/Ni^{3+} redox couple; high surface area
	Fe_3O_4 in Au/chitosan-c-MWCNT/LOx composite [170]	Enzyme anchoring; catalytic enhancement; magnetic actuation
Amperometric (cont.)	rGO/SiO_2 nanospheres on CNT-PDMS [37]	Structural scaffolding; surface area enhancement
	Methacrylated hyaluronic acid hydrogel microneedles [34]	ISF extraction; diffusion control; mechanical compliance
	Au gel@polydopamine-grafted ferrocenecarboxaldehyde [58]	Signal amplification; biocompatible redox interface
	Emulsion-based porous BSA/AuNW nanocomposite [17]	Antifouling; conductivity enhancement
	Enzyme-loaded mesoporous silica (MCM-41/SBA-15) [127]	High-density enzyme encapsulation; controlled analyte access
	Boronic acid-functionalised MIL-100(Cr)-B [108]	Hierarchical porosity; specific enzyme binding; immobilization scaffold
	Ag NPs embedded in Ni-MOF matrices [109]	Percolation pathways; interfacial electron transfer
	UiO-66-NH_2 hosting electroactive dye tags [107]	Signal amplification via pore-concentrated electroactive species
	β-CD-rGO/GCE [217]	Encapsulation; host–guest selectivity coupled with rGO charge transport
	Surface-bound CDs on nanoporous gold [44]	Selectivity control; high interfacial area; host–guest affinity
	Pd^{2+}@P-CDP/COF nanocomposite [220]	Co-localized binding and catalytic sites; porous framework
	AuNP-doped sol–gel silica xerogel electrode [123]	Precursor-tuned analyte partitioning; enzyme stabilization; mediated electron transfer
Voltammetric	MXene–graphene/PPy-MIP composite [79]	Selective recognition; conductive scaffold; signal amplification
	MXene–graphene (MXG)/MIP layer [35]	Selective recognition; conductive scaffold; immobilization
	Cu(II)/rGO nanocomplex [40]	Non-enzymatic electrocatalysis; signal amplification
	VMSF on pre-treated GCE (dopamine) [131]	Electrostatic enrichment; antifouling; molecular sieving
	VMSF on 3D macroscopic graphene (uric acid) [134]	Molecular sieving; macromolecular exclusion
	NH_2-VMSF (amine-functionalised) [130,133]	Charge-selective enrichment; signal amplification
	PAA/NB/IL hydrogel [55]	Mechanical integrity; physiological compatibility
	Graphene–MWCNT modified GCE [203]	Synergistic conductivity; high electroactive surface area
	Methyldopa-based BCAA MIP [78]	Selectivity control; mechanical stability; biofouling resistance
	Polyethylene-*block*-polystyrene membrane [14]	Size-selective diffusion; permeability control
Potentiometric	Poly(aniline) + methacrylic acid MIP [72]	High affinity; electron transfer; selectivity control
ECL	GDH-functionalized VMSF on ITO [135]	Enzyme anchoring; electrostatic luminophore enrichment
**Electrical**		
FET	Graphene electrolyte-gated transistor with AuNPs/rGO gate [39]	Gate dielectric modulation; rGO-AuNP composite enhances field effect
OECT	n-type accumulation-mode OECT (lactate oxidase) [90]	Volumetric ion-to-electron transduction; enzyme immobilization in conducting channel
	Enzyme-functionalised PEDOT-based OECT [89]	Volumetric redox transduction; metabolite-driven channel doping modulation
Chemiresistive	Methacrylic acid/EGDMA MIP membrane [13]	Size-selective permeability; analyte-gated ionic transport
**Other**		
Piezoelectric	PAAm/PDMA hydrogel [56]	Mechanical-to-electrical transduction
Magnetic	Superparamagnetic Fe_3O_4 nanoparticles [168,169]	Analyte enrichment; field-directed assembly
Thermal (HTM)	MIP particles on PVC base support [80]	Selective recognition; thermal transduction
	Surface-immobilized nanoMIPs [83]	Selectivity control; thermal signal transduction; surface immobilization
